# Maternal Postpartum Distress and Childhood Overweight

**DOI:** 10.1371/journal.pone.0011136

**Published:** 2010-06-30

**Authors:** Teresa A. Ajslev, Camilla S. Andersen, Katja G. Ingstrup, Ellen A. Nohr, Thorkild I. A. Sørensen

**Affiliations:** 1 Institute of Preventive Medicine, Copenhagen University Hospital, Copenhagen, Denmark; 2 Department of Epidemiology, Institute of Public Health, University of Aarhus, Aarhus, Denmark; Mayo Clinic, United States of America

## Abstract

**Objective:**

We investigated associations between maternal postpartum distress covering anxiety, depression and stress and childhood overweight.

**Methods:**

We performed a prospective cohort study, including 21 121 mother-child-dyads from the Danish National Birth Cohort (DNBC). Maternal distress was measured 6 months postpartum by 9 items covering anxiety, depression and stress. Outcome was childhood overweight at 7-years-of age. Multiple logistic regression analyses were performed and information on maternal age, socioeconomic status, pre-pregnancy BMI, gestational weight gain, parity, smoking during pregnancy, paternal BMI, birth weight, gestational age at birth, sex, breastfeeding and finally infant weight at 5 and 12 month were included in the analyses.

**Results:**

We found, that postpartum distress was not associated with childhood risk of overweight, OR 1.00, 95%CI [0.98–1.02]. Neither was anxiety, depression, or stress exposure, separately. There were no significant differences between the genders. Adjustment for potential confounders did not alter the results.

**Conclusion:**

Maternal postpartum distress is apparently not an independent risk factor for childhood overweight at 7-years-of-age. However, we can confirm previous findings of perinatal determinants as high maternal pre-pregnancy BMI, and smoking during pregnancy being risk factors for childhood overweight.

## Introduction

Childhood overweight is increasing worldwide and the reason for the increase remains uncertain [1]–[3]. Previous studies have suggested that perinatal determinants are of aetiological importance, for example: maternal pre-pregnancy BMI, gestational weight gain, gestational diabetes, and smoking during pregnancy which are now established risk factors for childhood overweight [1], [2], [4]–[9]. In addition, breastfeeding has been found protective for overweight in childhood [4], [10], [11]. Childhood overweight is associated to later morbidity [12] and can have long-term psychosocial consequences for the child, e.g. stigmatization, discrimination and depression [2].

Parental neglect has previously been associated with overweight in childhood and early adulthood, OR 1.6, 95% CI [1.1, 2.1] and OR 7.1, 95% CI [2.6, 19.3] respectively [13]–[15]. Distressed mothers are perhaps at increased risk of neglecting their children, thus maternal psychological wellbeing in the postpartum period may also be a possible early determinant of childhood overweight.

The postpartum period contains many challenges for the mother. Hormonal changes, weight changes, and disturbed sleep are physical changes after giving birth, which affect mood and feelings of stress and which are also related to postpartum depression [16]. A meta-analysis found that 10–15% of mothers had signs of postpartum depression 1–2 months after giving birth [17]. Postpartum depression is associated with increased irritability misdirected at the child and reduced mother-child interaction [15], [18]–[21]. The mother-child relation and early attachment are of importance for the overall development and psychological wellbeing of the child [19], [22]–[24]. A cross-sectional study found that attachment styles differed among mothers of obese and lean children [23]. This was not confirmed in a cross-sectional study by Stenhammer et al; however they found that family stress, especially high maternal stress scores on the SPSQ-stress scale, increased the risk of both overweight and underweight among Swedish children [25]. Furthermore, a recent cross-sectional study by Surkan et al. found an increased risk of overweight in offspring of mothers with higher maternal depression scores [26]. On this background, we hypothesize that maternal postnatal distress may influence the risk of later development of overweight and obesity in the offspring, and we speculate that the mechanisms could be the following ones: Maternal distress may influence the stress level of the infant through direct and indirect mechanisms of behavioral and hormonal origin. Changes in behavior may include psychological neglect or changes in feeding practices (food availability). The hormonal influence may be due to changes in stress hormones, which could be mediated through breastfeeding. Thereby maternal distress can contribute to the metabolism and growth of the infant through changes in hormonal and endocrine responses, thus leading to overweight. Charmandari et al. described the complex regulation of the hypothalamic-pituitary-adrenal (HPA)-axis during acute and chronic stress in both child and adulthood, where adaptational changes in behavior and metabolism might become chronically [27]. This hypothesis is comparable with the general conceptual framework of developmental origin of health and disease, and it also provides the rationale for distress changes in the early perinatal period to be of importance for later obesity (23–26). Thus the aim of this study was to investigate maternal feelings of distress in the early postpartum period as an underlying independent risk factor for childhood overweight.

## Materials and Methods

The study population consisted of 21 121 mother-child-dyads participating in the Danish National Birth Cohort (DNBC). Over 100 000 children born from 1997–2002 were enrolled in the original birth cohort (24).

The mothers were interviewed by telephone, twice during pregnancy (Interviews 1 and 2), and twice after pregnancy when the children were 6, and 18 months old, respectively (Interviews 3 and 4). The cohort has been thoroughly described elsewhere [28]. The 7-year-follow-up is ongoing and the first wave used in the present study included 40 640 children. The follow-up was carried out by a questionnaire, filled in by the parents by Internet or in paper format.

**Figure 1:**
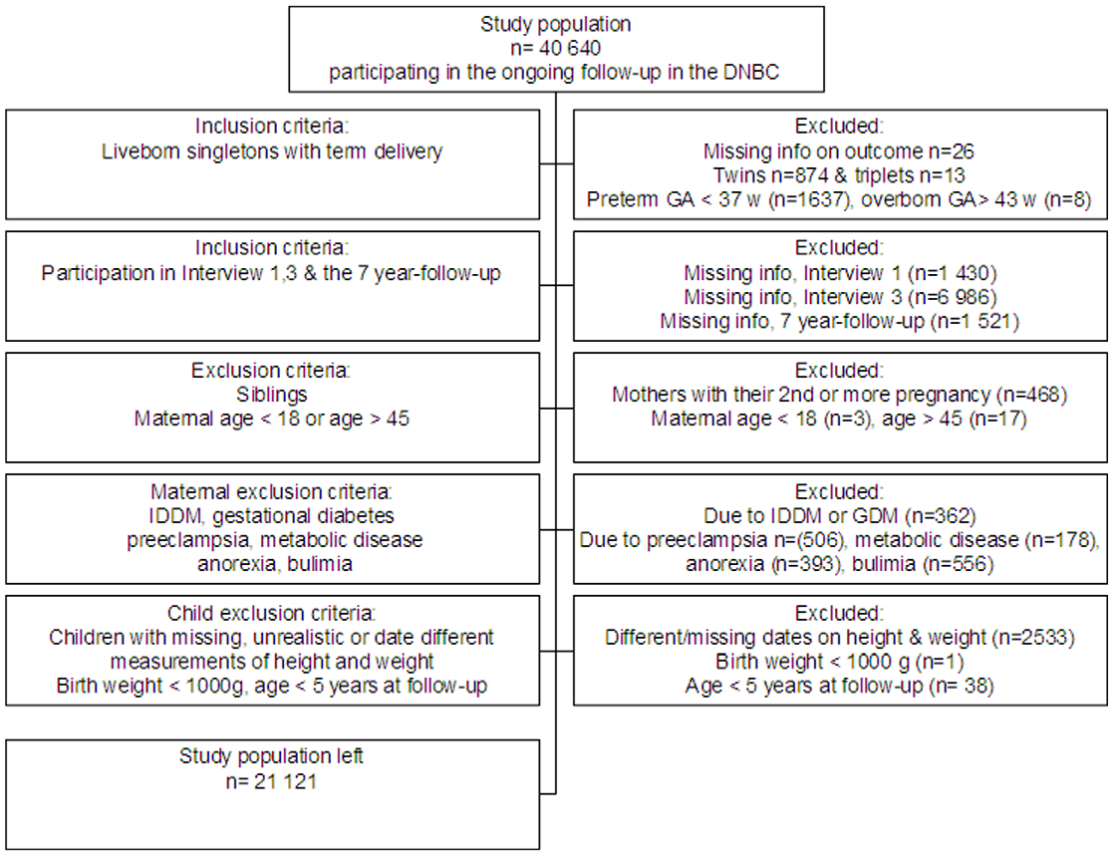
Flow chart of the study population.

In this study we included term, live born singletons, where the parents at the 7-year follow-up stated that measurements of height and weight were carried out on the same date. Younger siblings also included in the birth cohort were excluded. Furthermore, mothers should have participated in both Interview 1 and 3, and they should have provided information on pre-pregnancy height and weight and about gestational weight gain. Mothers with gestational diabetes, preeclampsia, metabolic diseases or having a former or current eating disorder were excluded. Mothers <18 or >45 years were also excluded from the analyses as were children with very low birth weight (<1000 g). In case of missing data or if the mothers answered, “do not know” or “do not wish to answer” in any of the 9 distress questions in Interview 3, they were also excluded. This gave a final study population of 21 121 mother-child dyads. The inclusions and exclusions are shown in Figure 1.

### Exposure variables

**Figure 2:**
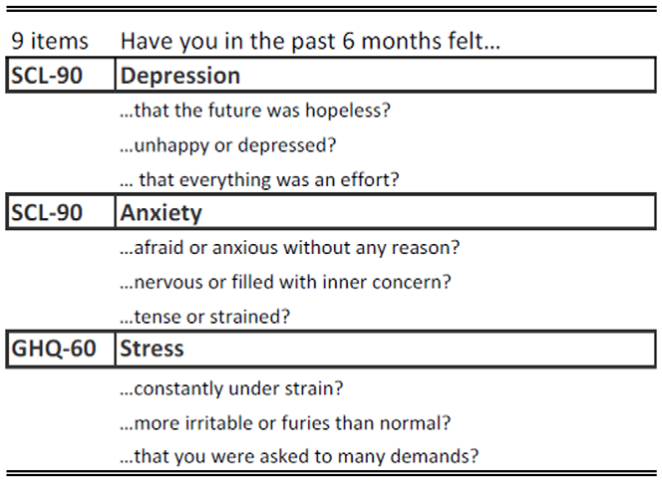
Distress questions. The questions about depression and anxiety were taken from the Symptom Distress Checklist (SCL-90). The questions about stress came from the General Health Questionnaire (GHQ-60).

Maternal postpartum distress was assessed by 9 questions asking the mothers about feelings of anxiety, depression and stress in Interview 3, carried out six months postpartum. Each question presented an item and was taken from two earlier validated questionnaires i.e., the Symptom Distress Checklist (SCL-90) [29] and the General Health Questionnaire (GHQ 60) [30]. The questions asked are shown in Figure 2. The mothers could answer “no”, “a little” or “a lot”, which refers to a 3-point likert scale. The answers were coded; “no” = 0, “a little” = 1 and “a lot” = 2. Preliminary analyses found Chronbach's alfa >0,81 for the nine items, which indicates medium to high internal reliability. Therefore, an overall estimate of the mother's **Distress** was generated, where the scores from the nine items were summed together. This gave a distress score ranging from 0 to 18 points. Variables covering **Anxiety** (score 0–6), **Depression** (score 0–6) and **Stress** (score 0–6) were constructed as three different total scores and the scores from the three items under each area were summed into a total score.

### Outcome variable

The outcome measure was childhood overweight at 7years of age. Body Mass Index (BMI =  weight/(height (m)^2^) were calculated from the parents reports on the child's height and weight. The age of the child at follow-up ranged from 5–8 years. We therefore used international age and sex specific cut-off scores for childhood overweight, which correspond to a BMI ≥ 25 as an adult if the child remains at the same growth curve [31]. The analyses were also adjusted for the age of the child at follow-up. International cut-off scores for obesity (BMI ≥30) and thinness (BMI <18.5) were used in complementary analyses to investigate non-linear relations [32].

### Covariates

We used information about maternal age, socioeconomic status, parity, smoking status and pre-pregnancy weight and height from Interview 1. Socioeconomic status was categorized into high, medium and low socioeconomic status. The women classified with high socio-economic status had a job that required a higher education, generally four years beyond high school [33]. Parity was coded into either primiparous (0) or multiparous (1–8^th^ previous births). The smoking variable was categorized into nonsmokers, smoking between 1–10 cigarettes per day or above 10 cigarettes per day (11+). Maternal pre-pregnancy BMI was coded into five categories (BMI <18.5, 18.5≤ BMI <25, 25≤ BMI <30, 30≤ BMI <35, and BMI ≥35). From Interview 3, we used information on maternal gestational weight gain and breastfeeding. Maternal gestational weight gain was coded into five categories <10 kg, 10–15 kg, 16–19 kg, 20–25 kg and >25 kg. Breastfeeding was categorized in three categories 0–14 weeks, >14–21 weeks, or >21 weeks. The lowest category also included women who never breastfed. From Interview 4, information on the father's height and weight as well as infant weight at 5 and 12 month was collected. Paternal BMI was calculated and used as a continuous variable. From the Danish National Birth Registry, information on pregnancy outcome, child sex, birth weight, and gestational age at birth were obtained.

### Statistical methods

By use of Chi-square and Student T-tests we examined group differences between normal weight and overweight children and between the three maternal distress variables across categories as pre-pregnancy BMI, gestational weight gain, parity, paternal BMI, birth weight, breastfeeding, smoking, and sex. We then used multiple logistic regression analyses to estimate the associations between the combined maternal distress report, entered as a continuous variable, and childhood overweight. Interactions between distress exposure and other covariates in their relation to the outcome were investigated, but no interactions were found. In the adjusted analyses we chose two different strategies. In the 1^st^ adjusted model we included confounders chosen a priori which were; maternal age, socioeconomic status, smoking, parity, pre-pregnancy BMI, gestational weight gain and covariates as paternal BMI, child sex, gestational age at birth, and age at 7-year-follow-up. In the 2^nd^ adjusted model we also included possible confounders as birth weight, breastfeeding and the child's weight at 5 and 12 months of age. Finally, we also performed the adjusted analyses for each sex separately. We used Intercooled STATA version 9.2 (Stata Corp, Texas) for all analyses.

### Ethics statement

The Danish National Committee on Biomedical Research Ethics approved the establishment and the great data collection of the DNBC after a thorough assessment. Written informed consent was obtained from each of the participants at the time of enrollment. The mothers have been able to withdraw from the DNBC at any time [34]. The Danish Data Protection Agency and the Institutional Board Committee of the DNBC approved this study's objective.

## Results

The prevalence of childhood overweight was 8,8% in boys (n = 903), [mean BMI 15.7±SD 1.6] and 10,5% in girls (n = 1133)[mean BMI 15.7± SD 1.7]. The maternal distress score ranged from 0 through 18 with a mean score of 2.1, 95% CI [2.06–2.13], and 7300 mothers reported no distress at all. Minor differences were found in the mean reports of anxiety (mean 0.86, 95% CI [0.84–0.87]), depression (mean 0.59, 95% CI [0.57–0.60]) and stress (mean 0.66, 95% CI [0.64–0.67]) respectively. Significant differences between normal weight and overweight children were found across nearly all covariates, shown in Table 1. Overweight in childhood was more often seen in children of mothers, with higher pre-pregnancy BMI (crude, OR 1.85, 95% CI [1.75–1.95]) or higher gestational weight gain (crude OR 1.10, 95% CI [1.06–1.15]), who smoked during pregnancy (crude OR 1.46, 95% CI [1.36–1.57]), had higher parity (crude OR 1.16, 95% CI [1.06–1.27]), or shorter duration of breastfeeding (crude OR 1.18, 95% CI [1.11–1.25]). Also, women of overweight children seemed to be of lower social status.

**Table 1:** Distribution of covariates for normal weight and overweight children, respectively.

	Normal weight		Overweight		
	%	mean, SD	%	mean, SD	
Subgroups of covariates	n = 19036		n = 2035		p-value
**Maternal age** (years)		30.7 (4.1)		30.6 (4.2)	<0.020
age 18–24	7.0		8.5		
age 25–29	38.6		37.1		
age 30–34	39.0		39.8		
age≥35	15.4		14.7		
**Socioeconomic status**					<0.005
High	55.5		45.9		
Middle	37.4		43.2		
Low	7.2		10.9		
**Prepregnant BMI** (kg/m^2^)		23.1 (3.6)		25.3 (4.7)	<0.005
<18,5	4.3		1.7		
18,5–24.9	72.6		54.4		
25–29.9	17.9		29.8		
30–34.9	4.2		9.6		
≥35	1.1		4.5		
**Gestational weight gain** (kg)		15.2 (5.2)		15.7 (6.1)	<0.005
<10 kg	10.9		13.5		
10–15.9 kg	46.9		39.3		
16–19.9 kg	21.7		21.4		
20–24.9 kg	14.1		16.1		
≥25 kg	6.4		9.8		
**Smoking**					<0.005
no smoking	78.2		69.0		
1–10 cigarettes pr day	17.4		22.8		
>11 cigarettes pr day	4.5		8.2		
**Parity**					<0.005
primiparous	43.4		39.8		
multiparous	56.6		60.2		
**Breastfeeding**					<0.005
<14 weeks	27.1		34.3		
14–22 weeks	40.0		35.2		
>22 weeks	32.9		30.5		
**Birth weight**					<0.000
<2500 g	0.8		0.5		
2500–4000 g	74.4		65.5		
>4000 g	24.8		34.1		
**Paternal BMI**		24.9 (3.0)		26.5 (3.6)	<0.005

Overweight children had higher birth weight and higher weight and height at 5 and 12 months of age. No overall significant group differences were found between maternal distress reports of normal weight and overweight children (results not shown). Reports of overall distress and of anxiety, depression, and stress varied across the covariates shown in Table 2. Mothers with high distress reports were overall of low or very high age, had lower socioeconomic status, higher gestational weight gain and were more often smokers. Primiparae were more likely to have feelings of anxiety and depression and multiparae reported higher stress amounts. Mothers with shorter duration of breastfeeding had higher reports of distress as well as stress, anxiety and depression.

**Table 2:** Distribution of covariates in relation to maternal postpartum distress scores.

	Distress, mean 2.1±SD 2.6				Anxiety, mean 0.9±SD 1.1				Depression, mean 0.6±SD 1.0				Stress, mean 0.7 ±SD 1.0			
	Score (0)	(1–9)	(10–18), %		Score (0)	(1–3)	(4–6), %		Score (0)	(1–3)	(4–6), %		Score (0)	(1–3)	(4–6), %	
Subgroups of covariates	n = 7300	n = 13362	n = 459	p-value	n = 9929	n = 10636	n = 556	p-value	n = 13585	n = 7078	n = 458	p-value	n = 13035	n = 7601	n = 485	p-value
**Maternal age** (years)				<0.000				<0.000				<0.000				<0.001
age 18–24	5.9	7.8	9.8		6.2	7.9	10.3		6.4	8.6	8.7		6.8	7.7	7.2	
age 25–29	37.8	38.8	37.0		37.6	39.3	38.5		38.1	39.0	39.1		39.3	37.2	33.6	
age 30–34	40.4	38.5	35.7		40.1	38.3	36.3		40.0	37.7	33.4		39.1	39.1	39.6	
age ≥35	16.0	14.9	17.4		16.2	14.6	14.9		15.6	14.7	18.8		14.8	15.9	19.6	
**Socioeconomic status**				<0.000				<0.001				<0.000				<0.078
High	69.4	69.5	65.7		69.4	68.7	62.9		70.1	67.2	61.2		68.7	69.4	66.9	
Middle	27.9	27.6	30.0		27.8	28.3	31.7		27.4	29.2	33.0		28.5	27.4	29.2	
Low	2.7	3.0	4.3		2.8	3.0	5.4		2.5	3.6	5.7		2.8	3.3	3.9	
**Prepregnant BMI** (kg/m^2^)				<0.510				<0.063				<0.528				<0.036
<18,5	4.2	4.0	4.2		4.0	4.1	3.6		4.0	4.1	3.9		4.0	4.0	5.0	
18,5–24.9	71.3	70.6	70.8		71.9	70.0	69.0		71.2	70.1	70.5		70.8	70.9	71.1	
25–29.9	18.3	19.4	18.1		18.1	19.8	20.3		18.6	19.8	18.8		18.8	19.5	18.0	
30–34.9	4.9	4.6	5.9		4.7	4.6	5.8		4.8	4.6	5.9		5.0	4.3	5.2	
≥35	1.4	1.4	1.1		1.3	1.5	1.1		1.4	1.5	0.9		1.5	1.3	0.8	
Gestational weight gain (kg)				<0.000				<0.000				<0.002				<0.034
<10 kg	11.7	10.8	13.7		11.4	10.9	11.5		11.2	11.0	13.8		11.2	11.1	11.6	
10–15.9 kg	47.8	45.5	39.2		47.3	45.4	41.0		47.0	45.0	38.7		46.5	45.7	41.7	
16–19.9 kg	21.0	22.1	22.0		21.7	21.8	20.3		21.4	22.3	22.3		21.5	22.3	19.8	
20–24.9 kg	13.5	14.7	15.7		13.5	14.9	16.7		13.9	14.9	15.7		14.3	14.2	16.9	
≥25 kg	6.1	6.9	9.4		6.1	7.0	10.4		6.5	6.8	9.6		6.5	6.7	10.1	
**Smoking**				<0.000				<0.000				<0.000				<0.000
no smoking	78.8	76.1	71.4		79.1	76.0	68.2		79.0	74.6	67.3		78.6	75.9	64.3	
1–10 cigarettes pr day	17.0	18.5	21.4		16.8	18.7	22.3		16.9	19.6	22.5		17.2	18.6	26.0	
>11 cigarettes pr day	4.2	5.4	7.3		4.1	5.2	9.5		4.2	5.8	10.3		4.2	5.6	9.7	
**Parity**				<0.000				<0.000				< 0.003				<0.003
primiparous	42.5	43.8	44.6		40.2	45.4	49.6		42.2	44.5	45.4		44.0	41.7	40.4	
multiparous	57.5	56.2	55.4		59.8	54.6	50.4		57.8	55.5	54.6		56.1	58.3	59.6	
**Breastfeeding**				<0.000				<0.000				<0.000				<0.000
<14 weeks	26.3	28.3	34.3		26.2	28.5	43.0		25.7	30.6	47.8		26.9	28.4	40.2	
14–22 weeks	40.6	38.7	35.9		40.5	39.0	33.8		40.8	38.0	26.9		40.8	37.9	33.2	
>22 weeks	33.2	33.0	29.8		33.3	32.6	23.2		33.5	31.5	25.3		32.3	33.7	26.6	
**Birth weight**				<0.007				<0.001				<0.013				<0.112
<2500 g	0.5	0.9	1.5		0.5	1.0	1.1		0.6	0.9	1.5		0.7	0.8	1.7	
2500–4000 g	73.3	73.7	72.8		73.2	73.8	75.2		73.6	73.7	70.3		73.7	73.3	72.2	
>4000 g	26.2	25.5	25.7		26.3	25.3	23.7		25.8	25.4	28.2		25.6	25.9	26.2	

Results from the multiple logistic regression analyses are shown in Table 3. The unadjusted odds ratio for childhood overweight with one point increase in distress reports was OR 1.01, 95% CI [0.99–1.03], (p>0.05)[distress range 0–18]. No significant changes of the estimates were found when adjusting for covariates in any of the models. The logistic regressions for sex separately showed minor different tendencies between the associations of stress and overweight and between anxiety or depression and overweight (results not shown), but there were no overall significant sex differences. We also compared the group of mothers with a distress score of zero (n = 7300) with the rest of the study population. These children's risk of overweight were found to be OR 0.97, 95% CI [0.88–1.06].

**Table 3:** Maternal distress and risk of childhood overweight.

	Unadjusted		Model 1		Model 2	
Subjects n = 21 121	OR	95% CI	OR	95% CI	OR	95% CI
Distress, score (0–18)	1.01	[0.99–1.03]	1.00	[0.98–1.02]	1.00	[0.98–1.03]
Depression, score (0–6)	1.02	[0.98–1.07]	1.01	[0.96–1.07]	1.02	[0.96–1.08]
Anxiety, score (0–6)	1.02	[0.98–1.07]	1.00	[0.96–1.06]	1.02	[0.96–1.07]
Stress, score (0–6)	1.00	[0.96–1.05]	0.99	[0.94–1.05]	0.99	[0.93–1.05]

Odds Ratios (OR) are presented with 95% confidence intervals (CI).

Distress is the summed score of the 9 items covering depression, anxiety and stress.

**Model 1**. OR are adjusted for maternal age, socioeconomic status, parity, smoking, pre-pregnancy BMI, gestational weight gain, paternal BMI, gestational age at birth, child age at 7 years follow-up, and sex.

**Model 2**. OR are adjusted as model 1, and also for birth weight, breastfeeding and child weight at 5 and 12 months-of-age.

In supplementary analyses, we investigated possible relations to either obesity or thinness, by using cut-off scores for obesity (n = 211) and thinness (n = 2731). The analyses showed no significant association between maternal distress and childhood obesity OR 1.02, 95% CI [0.99–1.06], neither for depression, anxiety and stress reports separately. When excluding the group of thin children (n = 2731), from the logistic regression analyses there were also no indications of any associations between distress and childhood overweight, neither were there any relations to obesity. Furthermore, no associations were found between maternal distress and risk of thinness OR 1.00, 95%CI [0.98–1.01].

We also investigated the prevalence of overweight in children of mothers, who did not participate in Interview 3. This prevalence was 9.8% in contrast to 9.7% in those who did participate in Interview 3 (results not shown). Also, we repeated the adjusted analyses controlling for distress reports during pregnancy. This did not change any of the estimates.

## Discussion

In this prospective cohort study, associations between maternal postpartum distress and childhood overweight were investigated. The results showed that maternal postpartum distress was not an underlying independent risk factor for childhood overweight at 7-years-of-age. With the study design of prospectively collected information, we had an excellent opportunity to investigate these associations without the risk of recall bias. Also, we found no significant differences between genders, and neither did we find any associations between reports of anxiety, depression, and stress separately and childhood overweight. In addition, our study confirms the previous findings of perinatal determinants being associated with childhood overweight including maternal smoking during pregnancy, high maternal and paternal preconception BMI, maternal gestational weight gain and a protective effect of breastfeeding.

Maternal distress might lead to parental neglect, which was earlier found related to childhood overweight [13]–[15]. In contrast to our findings, a just published cross-sectional study by Stenhammar et al. found that maternal stress reports were related to both childhood over- and underweight [25]. In that study, adjustment for maternal pre-pregnancy BMI and smoking was not carried out and follow-up was carried out at a different point in time during childhood, which complicates the comparison of the two studies. The cross-sectional study by Surkan et al. [26] found a relation between maternal depressive symptoms and childhood overweight at 6–24 months of age but also here our study differs. Firstly, the two studies used different subscales for measuring postpartum distress, and the study by Surkan et al. only measured depression. Secondly, they did not adjust for maternal pre-pregnancy BMI [2], gestational weight gain [7], and paternal BMI [2], which are established perinatal risk factors for childhood overweight. Finally, our study had a longer follow-up. We measured the prevalence of childhood overweight at 7 years of age, which gives us knowledge of an association at that age. But since childhood overweight is a changing condition, some may have been overweight earlier in life but not at the specific time of measurement.

We investigated a period of infant distress exposure from birth until 6 months postpartum. The original neglect hypothesis found associations between neglect in school age and overweight in early adulthood [13], but the associations have also been seen in preschool children [14]. It is possible that the child can adapt to some maternal distress, but associations with long-term exposure could be different. We repeated the adjusted analyses controlling for prenatal distress reported during pregnancy, but this did not change our findings. Previous studies have investigated several kinds of psychosocial stress of the child and subsequent overweight. These studies investigated neglect [13]–[15], [35], [36], child sexual abuse [37], posttraumatic stress [38] and attachment-style [23] and they found associations to either overweight or obesity. However, these studies all had follow-up in adulthood, except for one of the neglect studies [14]. Maternal postpartum distress is only a proximal measure of the child's exposure to psychosocial stress, which could explain our negative findings. Previous observations of associations between psychosocial stress of the child and later obesity could also be related to other determinants in or around the child. The study of posttraumatic stress by Perkonigg et al. actually did not find associations to overweight, but found associations to obesity only among females, OR 3.8 [95% CI 1.4, 10.7] [38]. This may suggest possible differences in the dose-response relations to overweight and obesity. However, our supplementary analyses only on obese children did not support an association. Earlier cross-sectional studies found that childhood overweight and maternal distress were coexisting, and explained by stressful family interactions such as mealtime difficulties in families with overweight children or adolescents [24]. But the causality chain between the two is unknown. Oberlander et al. suggested that the infant feeding practice or the mothers handling of the child could affect the early programming of the HPA-axis and thereby stress and appetite regulation [39]. We found no overall interaction with breastfeeding. The fact that the mothers who breastfed the shortest had the highest distress scores, could indicate that mothers who felt distressed discontinued breastfeeding. A reverse causation is also possible, implying that mothers developed distress because of failure to breastfeed. We used internationally validated questions from the GHQ-60 & the SCL-90 to measure maternal postpartum distress. [29], [30]. However, distress reports from the mothers were somewhat lower than expected, possibly for several different reasons. First, the willingness of the women to join the project may imply that they were more robust than those not willing to join. The reports of stress, anxiety, and depression were given retrospectively which could have induced recall bias. The recall period of 6-months differs from the original SCL-90 and GHQ-60 questions, where women should recall symptoms in the last week (SCL-90) or 14 days (GHQ-60). Furthermore, the original SCL-90 and GHQ-60 questions were asked at a five and four point likert scale, respectively, and our questions were asked in a three point likert scale. This could explain the relatively low proportion of distress reports seen in the cohort population with a medium of 2.1 [range 0–18]. With a medium to high Chronbach's alfa (>0.81) the reliability on the distress questions was however found to be good. Nonetheless, the possible measurement bias probably would be equal for all women and therefore not bias the estimates. The information on the children's height and weight was self-reported, but a preliminary (unpublished) validation of these measurements shows no systematic bias.

Given the cohort design with prospectively collected information and the very narrow confidence intervals, the present study provides good evidence of the absence of an association between early postpartum distress and the child's risk of overweight in later life. However, the population consisted of healthy Danish women in a welfare society with overall low levels of distress and thus a low exposure contrast. The associations may be different in other populations with a higher level of distress in the postpartum period.

Several mother-child dyads were excluded from the study population to reduce heterogeneity and to reduce bias because of missing and invalid measurements. We excluded mothers with previous or current eating disorders because this illness may have influenced the fetus and infant nutrient supply and thereby possibly the developmental origin of metabolic programming [40], [41]. Also, mothers with eating disorders may feel and report increased distress because of co-morbidity, which we also found (mean distress 3.4 in anorectic women, 3.3 in women with bulimia, and 2.1 in other women) and therefore, an observed increased risk for childhood overweight would have been difficult to interpret. Yet, we investigated associations between maternal reports of anorexia and bulimia and childhood BMI in the excluded population and we found no significant associations (results not shown).

The study population was compared with the total population participating in the 7-year follow-up. Significant but very small differences were found on several exposures. The included children had mothers with lower pre-pregnancy BMI and higher parity; they were of higher social status and had longer duration of breastfeeding (results not shown). Also, the children included in this study had slightly lower BMI at the 7-year-follow-up. There were no differences between maternal gestational weight gain, smoking habits or paternal BMI.

The proportion of women reporting high distress (10–18) in Interview 3 was 3.8% compared to 2.7% of the participating mothers in the 7-year-follow-up. Also, the mothers that we excluded had higher distress scores 6 months postpartum than the rest of the study population. Higher distress reports were seen in mothers delivering preterm, mothers with preeclampsia and mothers with twins and triplets, which actually support the trustworthiness of the mothers' distress reports.

When the DNBC was established, 30% of all eligible women were recruited. A study by Nohr et al. found that low participation to the DNBC did not induce bias to selected exposure-outcome associations when participants were compared to the entire eligible population [42]. The follow-up period has been long and attrition has reduced the cohort sample substantially since interview 1. Selection bias may be possible related to lack of compliance to the 6-month follow-up in distressed mothers. However, the supplementary analyses that we carried out did not suggest such bias.

### Conclusion

This cohort study found no indication of an etiological relationship between maternal postpartum distress and the development of childhood overweight at 7-years-of-age. In addition, our study confirm previous findings of high maternal pre-pregnancy BMI, high gestational weight gain, smoking during pregnancy and high paternal BMI being risk factors for childhood overweight in school age.
